# Storage Quality and Tocopherol Content of Crude Glandless Cottonseed Oil Under Accelerated Oxidation Conditions in Comparison with Commercial Cottonseed Oil

**DOI:** 10.3390/foods15101680

**Published:** 2026-05-12

**Authors:** Zhongqi He, Sunghyun Nam, Stephen I. Rogers, Scott M. Pelitire, Ocen M. Olanya

**Affiliations:** 1 USDA Agricultural Research Service, Southern Regional Research Center, 1100 Allen Toussaint Blvd., New Orleans, LA 70124, USA; sunghyun.nam@usda.gov (S.N.); scott.pelitire@usda.gov (S.M.P.); 2 USDA Agricultural Research Service, Eastern Regional Research Center, 600 East Mermaid Lane, Wyndmoor, PA 19038, USA; modesto.olanya@usda.gov

**Keywords:** acid value, cottonseed oil, glandless cotton, oxidation stability, peroxide value, shelf life, tocopherol

## Abstract

Cottonseed oil (CSO) is regarded as a nutritionally balanced oil, and is routinely used for food and cosmetics products. Autooxidation degrades oil storage quality and shortens the shelf life of oil products. In this research, crude CSO (c-CSO) from recently developed glandless cotton and commercially available refined CSO (r-CSO) from conventional glanded cotton were subjected to accelerated oxidation under storage at 60 °C in a convection oven for 45 days. Selective parameters (e.g., acid, peroxide, and anisidine values) and spectroscopic features (ultraviolet–visible absorptivity and Fourier transform infrared band intensity) were used to monitor the changes in the storage quality behaviors during the storage. The resulting data indicated that the specific values and the change trends of these parameters were not exactly the same between c-CSO and r-CSO. Generally, the c-CSO sample tended to show lower autooxidation degrees than r-CSO during storage. The content of tocopherol (a specific fat-soluble type of antioxidant compounds with a methylated phenolic ring) was 1016 and 23 mg kg^−^^1^, in the two oil samples, respectively. The decreasing trend of tocopherol content in c-CSO samples implied that the tocopherols played roles in slowing down c-CSO’s autoxidation process, thus increasing its shelf life. Information derived from this work would be helpful in the application of the new c-CSO as an effective antioxidant component in addition to conventional CSO’s nutrient values.

## 1. Introduction

Cotton (*Gossypium* spp.) is one of the most important crops grown in over 100 countries, covering an area of 33 million hectares and fulfilling around 31% of fiber needs for textile purposes [1]. Cottonseed is a major product after fiber of the cotton crop (*Gossypium* spp.) [2]. Cottonseeds can be utilized directly or further processed to produce fractions of linter, hulls, crude oil, meal (protein and carbohydrate), and waste [3]. However, the conventional cottonseed contains a yellow toxic chemical gossypol in its piment gland [2,4]. Gossypol gives an undesirable color to the oil and reacts with protein to reduce the nutritive value of cottonseed products [5]. The optimization of processing protocols and solvents has tried to remove the pigment from cottonseed so that it can be used in edible products without any adverse effect [6,7]. For food applications, the gossypol content of cottonseed products should be below 450 ppm, as required by the US Food and Drug Administration’s food standard, 600 ppm of the allowable limit, as per the United Nations Food and Agriculture Organization and the World Health Organization, or 200 ppm per the Chinese cottonseed food safety standards [8,9]. The refined cottonseed oil (CSO) products meet the standards, with gossypol content in the range < 100 ppm or even as low as 1 ppm for the cotton varieties and refining procedures [5,10,11]. On the other hand, alternative efforts have been made to develop new varieties with an ultralow content or free of gossypol, typically termed “glandless” cottonseed to distinguish it from the conventional “glanded” counterpart [12].

CSO is regarded as a nutritionally balanced oil due to its beneficial linoleic-to-oleic acid ratio, and is routinely used for food and cosmetics products [13,14]. Indeed, cottonseed, being the byproduct of cotton, is the first commercial-scale oilseed in modern times [10] and one of the five most widely used vegetable oils in the world [15]. To promote the food applications of novel glandless cottonseed, previously, we developed cottonseed butter products from roasted glandless cottonseed kernels with additional CSO [10], as plant-based butter (or spread) products have steadily increased in consumer popularity and have a lower environmental impact than dairy butter [16,17]. In plant butter products, oil content is a critical parameter which determines the product’s consistency and stability. For example, by mixing pecan flour with pecan oil in different ratios, Wagener et al. [18] found that pecan butters with 55–60% oil content were the most acceptable to consumers. Physically, pecan butter with 70% oil had the lowest viscosity and those with 50–55% oil had high viscosity. On the other hand, these high oil contents make the butter products more susceptible to rancidity, as lipid autooxidation is the main route for spoilage of edible oils and butter products [19]. Thus, artificial or natural antioxidants were tested in an attempt to retard oxidative changes in oils and oil-containing products [20,21].

While the oil content in the raw cottonseed kernels is around 35–39% of the kernel biomass [2], additional CSO is needed to make the cottonseed butter products with a total oil content of approximately 50% for appropriate textural properties [22,23]. We tested both commercially refined glanded CSO (r-CSO) and lab-made glandless crude CSO (c-CSO) [8,24]. While both butter products had similar structural and textural properties, the butter product with c-CSO showed a much longer (nearly 10 times) shelf life than the butter made with r-CSO. This difference could be attributed to the higher tocopherol contents in c-CSO than in r-CSO, as refining processes frequently accompany the loss of these antioxidant components [25]. Built on this hypothesis, this work was designed to conduct a comparative measurement of the changes in tocopherol contents and oxidative stability parameters of c-CSO and r-CSO under accelerated oxidative storage conditions [23,26]. It should be noted that the purpose of this work was not a one-to-one comparison of the relevant oil parameters between the two types of cottonseeds, as there were other differences in processing, storage history, and potential additives. The aim of this research was to evaluate the beneficiary stability behaviors and oxidation mechanisms of c-CSO in order to set a scientific foundation for the application of c-CSO as an effective antioxidant component, in addition to CSO’s nutrient values [27,28]. r-CSO was mainly included as a reference, as a common commercial CSO product.

## 2. Materials and Methods

### 2.1. Materials

Lab-made c-CSO through the mechanical pressing of glandless cottonseed was obtained as a gift from Cotton Inc. (Cary, NC, USA). The commercial conventional r-CSO product (Admiration Foods, Englewood, NJ, USA) was purchased from a local store. Hexanol, isopropanol, methanol, potassium hydroxide, and sodium carbonate were purchased from Fisher Scientific (Waltham, MA, USA). Phenolphthalein, and tocopherol standards were purchased from Sigma Aldrich (St. Louis, MO, USA).

### 2.2. Accelerated Storage Conditions

Per previous work [23,26], each oil sample (31 g) was placed in a 50 mL test tube, capped, and wrapped with aluminum foil to avoid light exposure. Sample tubes (36 in total) were then stored at 60 °C in a forced-air convection oven (Thermo Scientific, Waltham, MA, USA) for up to 45 days. Triplicate tubes were removed after 0, 5, 10, 20, 30, and 45 days, respectively. These samples were subjected to chemical analysis immediately or kept at −20 °C for later use.

### 2.3. Acid Value Measurement

For acid value determination [29,30], 2.00 g of samples were added into 20 mL of ethanol: ether (1:2, *v*/*v*) solution. About three drops of 1% phenolphthalein were added. Under continuous stirring, the sample was titrated with 0.1 M KOH until a pink color was observed. The acid value (I_KOH_, mg KOH g^−1^) was calculated using the following equation:

I_KOH_ = 56.1 × V × M/m,

where 56.1 represents the molecular weight of KOH, V represents the volume of KOH used for titration in mL, M represents the molarity of the KOH solution, and m stands for the mass of the sample (g).

### 2.4. Peroxide Value (PV) and p-Anisidine Value (pAV) Measurement

Peroxide value (PV) was then determined per the protocol of the AOCS Official Method Cd 8b-90 [31]. Data was expressed as milliequivalents of peroxide per kg of sample (meq kg^−1^) [32]. The pAV was determined according to the standard of AOCS Official Method Cd 18-90 using a spectrophotometer (Evolution 60S, Thermo Scientific, Waltham, MA, USA) at 350 nm [33].

The TOTOX value, a total oxidation parameter by considering both primary and secondary oxidation products [34,35], was calculated using the following equation:

TOTOX = 2 PV + pAV

### 2.5. High-Performance Liquid Chromatography (HPLC) of Tocopherol Compounds

The identification and quantification of tocopherol compounds were conducted based on the AOCS procedure Ce 8-89 [36]. Briefly, each oil sample (20.0 mg) was dissolved in 1.00 mL of hexane. These oil samples dissolved readily into hexane so that filtration was not required. The measurement (20 μL upload) was conducted with a Waters model 2695 HPLC pumping system with model 2475 fluorescence detector (excitation wavelength 290 nm, and emission wavelength 330 nm) and a Phenomenex Luna 5 μm Silica column (150 × 4.6 mm) (Milford, MA, USA) at room temperature (22 °C). The mobile phase was 99% hexane and 1% isopropanol, and was pumped at 1.0 mL min^−1^ [24]. A mixture of α-, β-, γ- and δ-tocopherol dissolved in hexane was used as the standard [37]. With a concentration around 6.00 mg L^−1^ for each tocopherol, 2 to 20 μL of the mixture was injected into the HPLC system to produce the linear standard curves for quantification (R^2^ ≥ 0.9900, n = 5, *p* ≤ 0.001). All standards and samples were protected from light until injection via storage in amber HPLC vials.

### 2.6. Ultraviolet/Visible (UV/Vis) Spectral Analysis

UV/Visible spectrophotometer (Evolution 60S, Thermo Scientific, Waltham, MA, USA) was used to obtain the spectra of CSO samples between 200 and 800 nm. The scan speed mode was set at a medium level with intervals of 1 nm. Standard 10 mm path length quartz cells were used for measurement [38]. To obtain the absorbance in the measurable range, the UV/Vis analysis was run with undiluted oil samples and then on samples diluted with hexane by factors of 10 to 5000 [39]. The UV/Vis spectral features of E_232_, E_270_ and E_4_/E_6_ were calculated from the absorbances at 232 nm and 270 nm and absorbance ratios of 400 nm and 600 nm, respectively [40,41].

### 2.7. Attenuated Total Reflectance–Fourier Transform Infrared Spectroscopy (ATR-FTIR) Spectroscopy

The spectroscopy analysis was performed by using a Vertex 70v spectrometer (Bruker Daltonics, Billerica, MA, USA) [2]. The ATR device was a MIRacle single-reflection ATR accessory (Pike Technologies, Fitchburg, WI, USA) with a diamond crystal plate as the reflector. Background spectra were collected before each sample measurement under identical conditions. The oil samples were deposited onto the ATR crystal surface. The spectra were collected over the range of 4000–600 cm^−1^ at 4 cm^−1^ resolution and with 16 scans. All spectra were normalized to the most intense band to enable comparison across samples and presented as absorbance values [42].

### 2.8. Data Treatment and Statistical Analysis

Triplicate accelerated storage experiments were conducted for both c-CSO and r-CSO samples. Data were presented as mean values of the triplicate tests. Differences between pairs of means were determined by Fisher’s Least Significant Difference procedure, and the subsequent assignment of letters for the grouping of means followed the method developed by Klasson [43]. The trendline fitting analysis of changes in tocopherols content over storage time was performed using the “format trendline” function in Microsoft Excel 2016.

## 3. Results and Discussion

### 3.1. Acid Value

The parameter of acid value represents the free fatty acid content in oil samples. The acid value (2.89 mg g^−1^) of c-CSO was slightly higher than that (2.70 mg g^−1^) of r-CSO (Table 1). The values were in the range of 0.37 to 11.50 mg g^−1^ for CSO samples, similar to previous reports in the literature [44,45]. Storage at 60 °C increased the values of both c-CSO and r-CSO. However, there was no significant (*p* > 0.05) difference in the acid values of c-CSO samples stored from 5 to 45 days. Their average value (3.18 mg g^−1^) accounted for about a 10.0% increase compared to the initial c-CSO sample. After 5 d storage, the acid value of r-CSO increased to 3.10 mg g^−1^ from its initial value of 2.70 mg g^−1^, representing a significant (*p* ≤ 0.05) 14.8% increase. The storage of samples for 10 d further increased the value to 3.31 mg g^−1^, which was not significantly (*p* > 0.05) different from those with longer storage times of 20, 30 and 45 d. The average (3.40 mg g^−1^) was a 25.9% increase from the 0 d value. During the accelerated oxidation at 60 °C, some oil molecules were expected to break down via the hydrolysis of triacylglycerols to release free fatty acids, consequently increasing the acid value of the test samples [46]. Thus, a high acid value implies that oil product was not kept under proper storage conditions. While increasing acid values are common for oil and butter products [26,47], the percentage increase in our CSO samples was much lower than other types of oil samples. For example, the acid value of crude walnut oil was reported to increase by 500% during a 45 d storage duration at 60 °C [26]. The acid values of peanut butter oil samples with 100% peanut or 90% peanut plus 10% ginger were increased from 1.90 and 2.17 mg g^−1^ to 2.43 and 3.20 mg g^−1^ (or 27.9 and 47.5%), respectively, after 8 weeks of storage at 40 °C [48]. The acid values of numerous sesame pastes varied, and they ranged from 1.52 to ~2.14, 2.45 to ~2.79 and 2.28 to ~2.47 mg g^−1^ when stored at 40 °C [49]. The relatively low increases in the acid values of our CSO samples during the accelerated storage conditions suggested that the breakdown of the triglycerols of CSO was a retarding process compared to the high susceptibility of other types of oils, as discussed later.

### 3.2. PV and pAV Parameters

The two parameters of PV and pAV were determined to evaluate the oxidative stability behaviors of c-CSO and r-CSO during the accelerated storage test at 60 °C (Table 1). PV parameter is a measurement of the concentration of hydroperoxide and peroxides produced during lipid oxidation [50]. Thus, measurement of this parameter is widely used to document the oxidative status of oil/lipid products, as peroxides promote the formation of aldehydes and other, smaller molecular mass compounds that negatively affect flavor [47]. The PVs were 59.3 and 44.8 meq kg^−1^, respectively, for C-CSO and r-CSO. These values were in the expected range of CSO products, as Liu et al. [51] reported the PV ranged from 5 to 70 meq kg^−1^ with non-transgenic CSO samples and from 17 to 350 meq kg^−1^ with transgenic CSO products. During the accelerated storage time, the PV of c-CSO decreased slightly, then increased, and decreased again to a final value of 22.9 meq kg^−1^, which was significantly (*p* ≤ 0.05) lower than the initial PV measurement. In contrast, there were no statistically significant (*p* > 0.05) differences in PV values among r-CSO samples during storage times. The PV fluctuation in c-CSO samples might be attributed to the high reactivity of hydroperoxides, which leads to their decomposition/consumption not always matching the lipid oxidation rates. Thus, as a precaution, lower PV readings should not necessarily be considered an indication of the good quality of oil samples [10]. In other words, this method alone is not an accurate measurement of the total deterioration of oil products [52].

For this reason, the parameter of pAV was also measured as this represents the decomposition of the hydroperoxides in oil products and was used as an empirical test for monitoring the secondary products of lipid oxidation [53]. The pAVs of c-CSO and r-CSO were 0.12 and 4.85, respectively. Although not exactly matching, these values were comparable to the literature values (1.80–2.15) of CSO, extracted by solvent extraction and cold-pressing [54]. Even though r-CSO showed a much higher pAV reading, both CSO samples met the pAV recommendation of <10^1^ for good-quality oil products [10,52]. Unlike the PV parameter, the pAV of c-CSO samples steadily increased with storage time while the pAV of r-CSO samples was also significantly (*p* ≤ 0.05) higher at a later storage phase (d 30 and 45) than that of the initial sample. The increase in pAV during accelerated storage suggests the accumulation of anisidine reactive compounds. The phenomenon of a steady increase in pAV was previously observed with soy, sunflower and olive oils [55]. On the other hand, lower and fluctuating pAV readings depending on storage were observed with plant butter products, such as sunflower and sesame pastes [49,56] and cottonseed butter [23]. These irregular changes, which were not observed in the CSO samples, might be due to the involvement of the phenolic carboxyl acid group in non-oil fractions in the butter products and the C=C bond structure involved in the formation of carbonyls [56,57].

**Figure 1:**
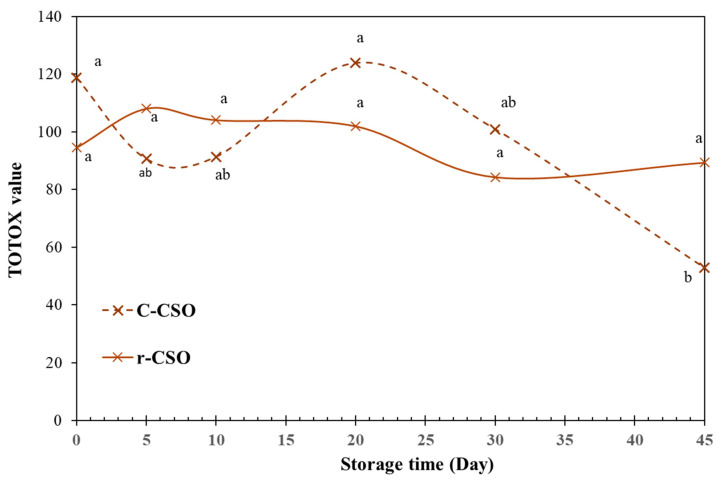
Changes in total oxidation parameter, TOTOX, of c-CSO and r-CSO samples over the accelerated storage time. Data are presented in the format of an average of triplicate experimental data. Different letters in the same curve indicate the values are significantly different over the storage period at *p* ≤ 0.05.

For a more comprehensive evaluation of the oxidation process, the TOTOX values over the storage time were calculated (Figure 1). These TOTOX values were in the range of refined cottonseed oil samples impacted by high-temperature storage. For example, Basturk et al. [58] reported continuously increased values of 79.8, 133.9 and 224.7 for a refined CSO sample at 7, 14, and 21 days of storage at 60 °C, although the initial value was low. However, unlike the CSO sample, the TOTOX value of our c-CSO fluctuated, but showed a general decreasing trend over the 45 days of storage. The value of r-CSO was also changing over the 45 days, but those changes were statistically insignificant (*p* > 0.05). As the TOTOX values combined evidence about the past history (pAV) and present state (PV) of oil oxidation [53], the impacts of the storage on the lipid oxidation (deterioration) of the c-CSO product seem to suggest a dynamic (present state) process over an accumulative (past history) process. In contrast, the observation of the r-CSO sample we tested implied more balanced dynamics among the present and past processes.

### 3.3. Identification and Quantification of Tocopherol Isomers

**Figure 2:**
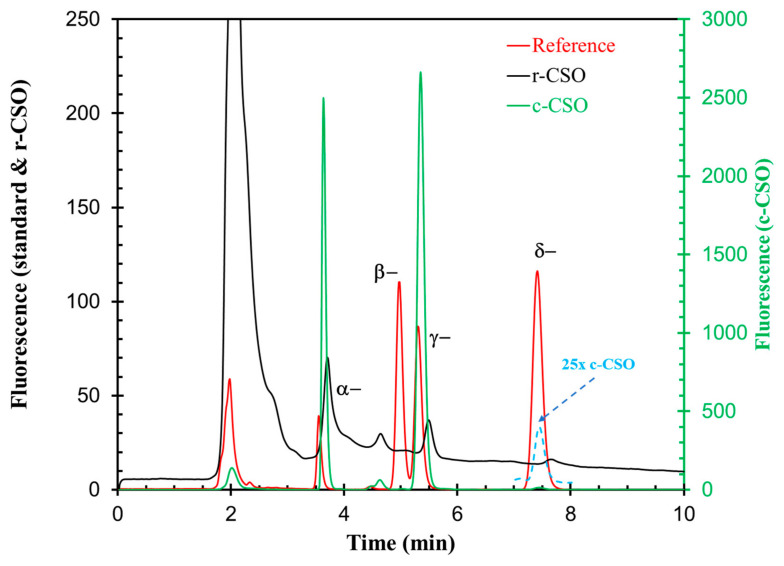
HPLC chromatograms of the α-, β-, γ-, and δ-tocopherol reference mixture (5 μL upload), c-CSO, and r-CSO samples prior to the accelerated storage experiment (20 μL upload). The blue dashed line between 7 and 8 min enlarges the fluorescence of c-CSO by 25-fold to show the δ-isomer signal of the c-CSO sample.

The representative HPLC chromatograms of the standard mixture and the two CSO samples are presented in Figure 2. The four tocopherol isomers were well separated under the HPLC operation conditions with retention times of 3.55, 4.98, 5.32 and 7.42 min, respectively. The chromatograms of c-CSO and r-CSO showed only two major peaks with retention times corresponding to those of α- and γ-tocopherols. In the meantime, it is notable that the peak intensity of the two tocopherols in r-CSO was very weak compared to the 100-fold strong ones of c-CSO. This phenomenon is consistent with the literature, which reported that a remarkable portion of tocopherol can be lost during processing [59]. Other two isomers, β- and δ-tocopherols were not detected or were very weak, as the two isomers in fresh hexane extracts of cottonseed kernel and butter products had concentrations of just about 1% of α- or γ-tocopherols [23,24]. It is apparent that the contents of β- and δ-tocopherols in the two current CSO samples were very close to or below the detection limit.

**Figure 3:**
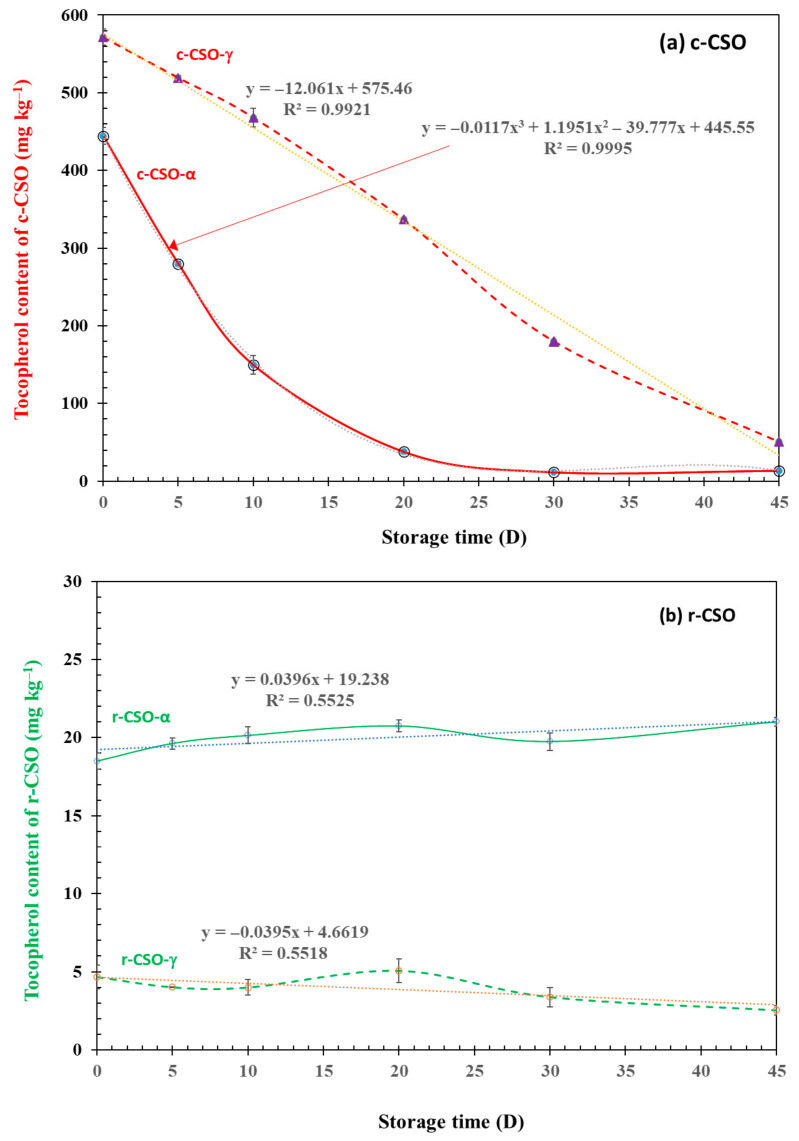
Changes in the tocopherols content of c-CSO (**a**) and r-CSO (**b**) samples during the accelerated storage time. Suffixes α and γ indicate α- and γ-tocopherol contents, respectively, in the two CSO samples. Data are presented as average (n = 3) with SD bars. The four equations show the pertinent trendlines of the tocopherol content of CSO samples versus their storage time.

Quantitatively, the initial contents of α- and γ-tocopherol were 444.4 and 571.4 mg kg^−1^ of the oil in c-CSO (Figure 3). The content of total tocopherol was around the typical 1000 ppm tocopherol in crude CSO [59]. While one third of tocopherol in crude CSO may be lost during the processing, the two tocopherol contents were just 18.5 and 4.66 mg kg^−1^ of oil in r-CSO. The tocopherol content in the r-CSO samples was also lower than some of the commercial CSO products in the literature, such as 180.0 mg tocopherol kg^−1^ of oil, as reported by Atta et al. [60], or the even higher values (460–1100 mg tocopherol kg^−1^ of oil) reported by Ahmed et al. [61] and Wen et al. [62]. The variation in the tocopherol contents among these refined CSO products could be attributed to the differences in the industrial oil production processes. Indeed, Wen et al. [62] observed that the tocopherol content varied from 85 to 1127 mg kg^−1^ in seven first-grade rice bran oil products, and from 1113 to 1279 mg kg^−1^ in four second-grade rice bran oil products.

With the increase in storage time, both α- and γ-tocopherol in the c-CSO sample rapidly decreased. However, the decreasing trends of the two isomers were different. γ-tocopherol content decreased linearly over the storage time (*p* < 0.001). On the other hand, the decrease in α-tocopherol content fitted a polynomial equation (*p* < 0.001). Although these values were low, the contents of the two tocopherol isomers in r-CSO were basically unchanged, showing a roughly flat linear line over the storage time even though the coefficient of determination (R^2^) was not significant (*p* > 0.05). The different change patterns between c-CSO and r-CSO were due to the impact of multiple factors, including but not limited to the oil source (type) and processing approach (refining). Previously, Yoon and Kim [63] reported that the tocopherol level of crude and degummed rice bran oil kept quite constant during 10 weeks storage at 50 °C; however, tocopherols in refined oil decreased slowly until 38 d of storage and then decreased sharply. Our observations seem to show a change in the opposite direction. One possible cause is the different types of oils. Another possible reason is that Yoon and Kim [63] tested fresh crude and refined rice bran oil samples while the cottonseed oils were stored at conventional temperatures for some time before the accelerated oxidation test at 60 °C. It should also be noted that, while tocopherols are primary phenolic compounds, other phenolic and non-phenolic minor lipid concomitants (e.g., gallic acid equivalent, carotene, and phytosterols) could also contribute to the antioxidant function of c-CSO, as in other seeds [25,34,40]. In addition, tocopherols are often categorized separately in food and lipid studies from other plant “phenolic compounds” (which are typically water-soluble) due to their oil-soluble nature.

### 3.4. UV/Vis Spectral Analysis

**Figure 4:**
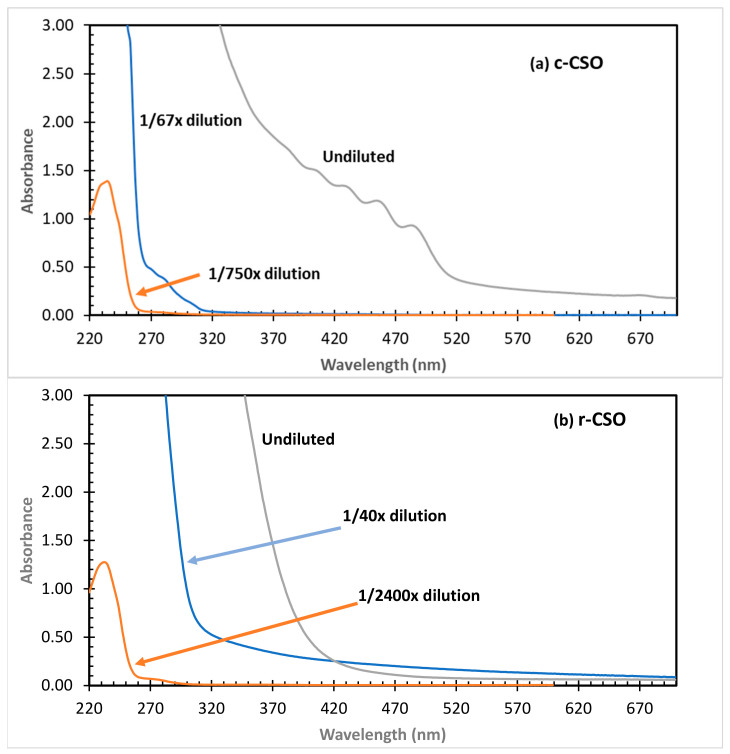
UV/Vis spectra of c-CSO (**a**) and r-CSO (**b**) samples. Undiluted and properly diluted oil samples in hexane were used in the analysis.

The UV/Vis spectra of the initial c-CSO and r-CSO samples are presented in Figure 4. This high dilution factor was needed to collect the absorbance data in the linear range. Lianhe et al. [39] reported the 1/1000 dilution to have a UV absorbance reading just below 3. The c-CSO sample showed UV-Vis spectral features with extremely strong peaks in the UV spectral region of around 232 and 270 nm, and multiple minor peaks in the visible spectral region between 400 and 500 nm. In general, good-quality oils have low absorption in the spectral range of 200 to 300 nm. On the other hand, UV-absorbing compounds can develop during some industrial processes or as a consequence of aging or bad storage [64]. The strong UV absorbance peaks in c-CSO and r-CSO samples indicate that there were abundant compounds with conjugated C═C and C═O double bonds (e.g., phenolics, aldehyde, and lipid chromophores) as intrinsic or oxidation products in these samples [19]. The minor visible spectral peaks indicate the presence of pigmented compounds in the crude oil samples, such as carotenoids with absorbance peaks at 400, 425, 455 and 480 nm [65,66]. Distinct from c-CSO, r-CSO samples showed monotonically decreasing curves with increasing wavelengths from 300 nm to 700 nm. This observation was attributed to the removal of the pigments in the refining process, similar to that of refined/bleached CSO samples in the literature [66]. On the other hand, the absorbances of the two UV spectral peaks were 2–5-fold stronger than the corresponding absorbance of c-CSO samples, implying that some UV-absorbing components were introduced during the refining process and/or as additives in the products [35].

The impacts of the accelerated oxidation storage were quantitatively evaluated by the three spectral parameters of I_232_, I_270,_ and the E4/E6 ratio (Table 2). The initial I_232_ values were 1.20 and 3.41 L g^−1^ cm^−1^, respectively, for c-CSO and r-CSO. The initial I_270_ readings were 0.0328 and 0.169 L g^−1^ cm^−1^, respectively, for c-CSO and r-CSO. The values of I_232_ and I_270_ are used for characterizing primary and secondary products of oil oxidation [19], and can be considered measures of conjugated dienes and trienes, respectively [40,64]. In 17 vegetable oil samples (olive, corn crystal, corn lecuir, sunflower, palm olein, soybean, linseed, cottonseed, sesame, rice bran, peanut, blending, wheat germ, safflower, rapeseed, castor and coconut oils), the I_232_ values varied from 0.16 L g^−1^ cm^−1^ (olive oil) to 7.80 L g^−1^ cm^−1^ (Safflower oil), and the I_270_ values from 0.08 L g^−1^ cm^−1^ (olive oil) to 5.25 L g^−1^ cm^−1^ (rice bran oil) [60]. The wide range of these values should not be attributed exclusively to the oil type. Post-extraction treatments and storage history/conditions might have also impacted them, as they were purchased from a local market in Egypt. In that work, the I_232_ and I_270_ values of CSO oil are 2.82 and 1.56, respectively. The I_232_ level of c=CSO and r-CSO was the same level as the commercial Egypt CSO sample, but the I_270_ levels of c-CSO and r-CSO were much lower than the reported Egypt CSO.

In this work, the I_232_ value of c-CSO increased by 31–37% after 20 d accelerated oxidation storage. There was no statistically significant (*p* > 0.05) change in the values of r-CSO samples. Similarly, the literature reported that the parameter of lab-produced oil products is more subject to the impacts of oxidation storage conditions than those commercial oil products [40,64]. The I_270_ values of both c-CSO and r-CSO samples steadily increased during storage times. While the initial readings were 0.0328 and 0.169 L g^−1^ cm^−1^, respectively, for c-CSO and r-CSO, their final readings increased to 0.161 and 0.338 L g^−1^ cm^−1^, representing about 5- and 2-fold increases. In previous studies, I_232_ was reported to be more than doubled, from 4.75 L g^−1^ cm^−1^ in canola oil to 10.24 L g^−1^ cm^−1^ after string in oven (63 °C) for 6 days [19]. In the same work, the I_232_ and I_270_ values were roughly doubled from 4.81 and 0.734 L g^−1^ cm^−1^ in canola oil to 10.58 and 1.60 L g^−1^ cm^−1^, respectively, after a 36 min microwave heating. Peanut oil samples showed I_232_ and I_270_ values of 0.012 and 0.015, which increased to 0.015 and 0.039 L g^−1^ cm^−1^ when peanuts were roasted at 80 and 200 °C, indicating greater oxidation of peanut oil at higher roasting temperatures [67]. While I_232_ and I_270_ are indications of primary (conjugated dienes) and secondary (conjugated trienes) products of oxidation [40,67], our data indicated that there a steady number of dienes and trienes were produced over the storage period of c-CSO. The number of primary dienes in the r-CSO sample was high but remained at a dynamically balanced level.

Another parameter, the ratio of absorbance at 400 and 600 nm (E_4_/E_6_), was also calculated (Table 2). While the absorbance at 400 nm decreased over the storage time (Figure 4), the E_4_/E_6_ value of c-CSO steadily increased. A great increase occurred in the first 5 d of storage with a reading that ranged from 6.91 on d 0 to 19.58 on d 5. After that, the reading gradually increased to around 25 after 30 d storage. The E_4_/E_6_ value of r-CSO also gradually increased from the initial values of 9.06 to 13.94 at the end of the accelerated storage on d 45. The difference in increase rates between the two CSO samples should be related to the lower pigment contents in the r-CSO sample, as visually observed, while no quantitative color data were measured. In agricultural research, this parameter is the relative measurement of aromaticity and the average molecular weight of organic matter components, such as soil-dissolved organic matter, tree extractive, and composts [68,69,70]. Lower E_4_/E_6_ values are typically attributed to higher average molecular mass components and more aromatic structures with higher degrees of condensation in the tested samples. For example, the parameter readings of humic acid samples with high and low molecular masses (i.e., > or <3 KD) were 3.6 and 15, respectively [71]. Thus, the increasing trends of the E_4_/E_6_ values in the two CSO samples might be considered one piece of evidence of the degradation (decomposition) of the CSO components over the storage time. However, more research is needed to confirm if this kind of relationship can be applied to the UV/Vis characterization of the oil products, as this parameter is rarely measured in the UV/Vis studies of food products [38].

### 3.5. ATR FTIR Spectral Changes

**Figure 5:**
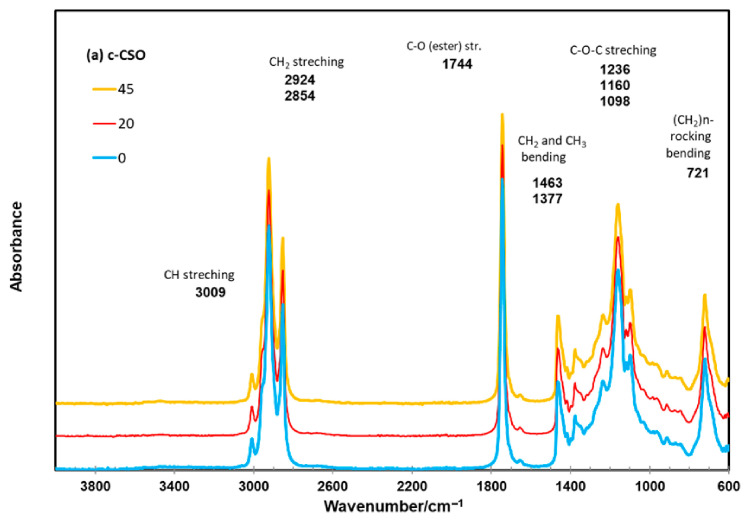

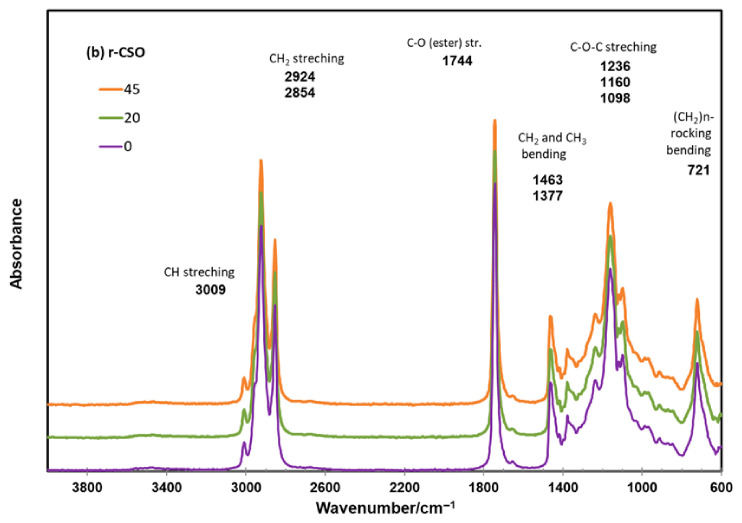
ATR FTIR spectra of c-CSO and r-CSO samples after accelerated storage for 0 (control), 20 and 45 d at 60 °C.

The ATR FTIR spectral features of c-CSO and r-CSO samples (Figure 5) are typical for the nonpolar (oily) fraction of cottonseed and CSO products [2,72,73]. In the FTIR features of vegetable oil samples [74,75,76], the minor but apparent peak at 3009 cm^−1^ is due to C–H stretching with the symmetric vibration of cis-olefinic double bonds (=CH). The multiple peaks from 2924 to 2854 cm^−1^ could be associated with the asymmetric and symmetric stretching vibration of the C–H of aliphatic CH_3_ and CH_2_ groups of triglycerides. The peak at 1744 cm^−1^ was due to the easter carbonyl (C=O) functional group of triacylglycerides. The region of other bond deformations and bending includes the small peaks in the region of 1463 to 1377 cm^−1^, which should be associated with the rocking vibrations of the C–H bonds of *cis*-disubstituted olefins and bending symmetric vibration of C–H bonds in the CH_2_ group. The peak at 1160 cm^−1^ flanking by minor 1236 and 1099 cm^−1^ peaks could come from the stretching and rocking vibrations of the –C–O ester group and -CH_2_– group. The peak at 721 cm^−1^ was associated with the out-of-plane vibrations of the –HC=CH− group of disubstituted olefins and overlapping CH_2_ rocking vibrations. In addition, a minor peak at 914 cm^−1^ could provide information on the *cis* double bonds and is involved in the transition between the second and third stages of oil oxidation, although it is not present in all oil samples [77,78]. Visually, the overall spectral features of the two types of CSO samples were quite similar over the accelerated oxidation storage times. Consistent with previous reports on FTIR investigations into the oxidative stability of edible oil products [23,78,79], this observation implied that the backbone C-functional groups (or major components) of these CSO samples did not change dramatically over the accelerated storage period.

**Figure 6:**
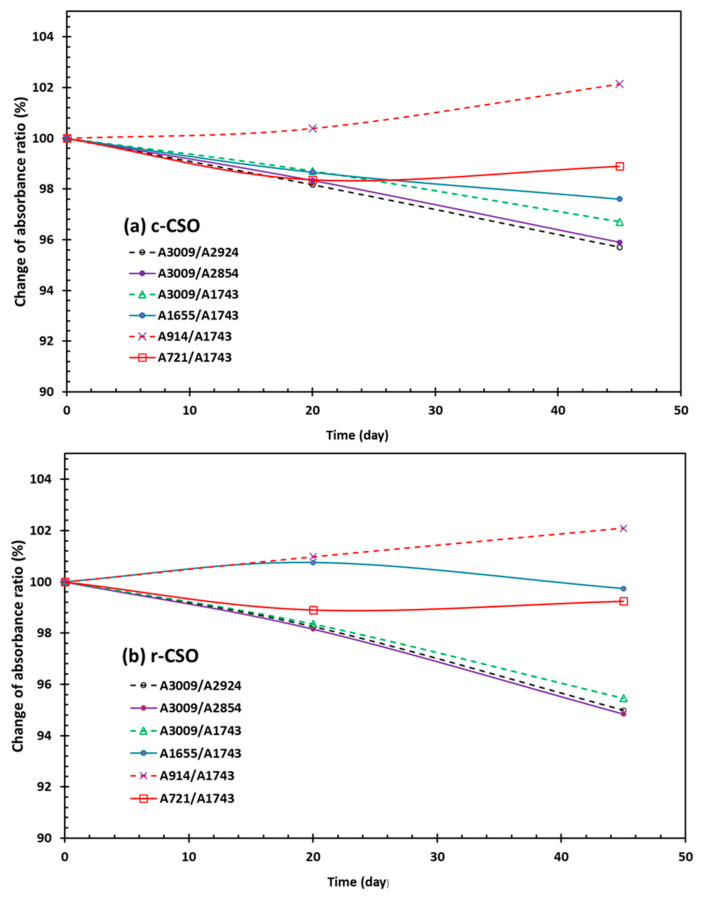
Relative changes (%) of selected ATR FTIR absorbance ratios of c-CSO and r-CSO samples during accelerated oxidation storage for 45 d at 60 °C.

However, focusing on the characteristic minor peaks in conjugated dienes and trienes FTIR analysis did provide useful information on the oxidative stability of vegetable oil samples impacted by processing and application factors [77,80,81]. For example, Shah et al. [82] reported that the band intensities at 3009, 2854, 1710, 1160 and 721 cm^−1^ matched the chemical parameter readings of the iodine value, PV, free fatty acids content, induction period, and saponification value of the CSO samples of five cotton varieties. Wen et al. [77] selected the four peaks at 3009, 1655, 914 and 721 cm^−1^ with ratios relative to the major peaks at 2924, 2854, or 1774 cm^−1^ to evaluate the FTIR changes in walnut oil samples over the accelerated storage. This is because these FTIR features are responsive to the oxidation products formed by polyunsaturated fatty acids, such as conjugated aldehydes and the conjugated double-bond system [77,78]. In this study, minor changes in the six FTIR absorbance parameters were observed during the accelerated storage period for both c-CSO and r-CSO samples (Figure 6). The general change in the trend of their readings was A_3009_/A_2924_ ≈ A_3009_/A_2854_ ≤ A_3009_/A_1743_ < A_1655_/A_1743_ < A_721_/A_1743_ < A_914_/A_1743_. The change in these parameters was more apparent than the CSO samples extracted from accelerated-storage cottonseed butter products [23]. The difference observed between the oil and butter samples could be attributed to the extra protection against oil oxidation offered by protein peptides and other minor bioactive compounds present in butter products [38,83,84]. Specifically, the ratio parameters of A_3009_/A_2924_, A_3009_/A_2854_ and A_3009_/A_1743_ showed a decreasing trend with increasing storage time. This decrease could be attributed to the reduction in 18:2 and 18:3 fatty acids content due to oxidation [85]. Indeed, the three parameters clustered together with percentage values around 95% at d 45 in the r-CSO sample, but displayed a wider but lower range of 95.7 to 96.7% in the c-CSO sample. Therefore, the changes in the three clustered parameters indicated that the relevant fatty acids in c-CSO were less oxidated than those in r-CSO. On the other hand, parameters A_1655_/A_1743_ and A_721_/A_1743_ were kept basically unchanged over the storage time. This observation differed from the complicated variation pattern of these ratios in walnut oil samples [77]. This is because walnut oil has a very high content of unsaturated fatty acid-conjugated aldehydes and the conjugated double-bond system, leading to active multiple-stage oxidation dynamics [77,86]. The A_914_/A_1743_ of both CSO samples indeed increased slightly to 102.0% on d 45. This increase is an indication of the transitive accumulation of the secondary oxidation products (e. g., conjugated alkenes) in the deepening (advanced) oxidation of CSO products [77,78]. These FTIR observations would be helpful in developing non-invasive techniques for rapid and accurate quality evaluations of CSO composition, oxidative stability, and shelf life [87].

## 4. Conclusions

The present study measured the changes in the multiple oxidative stability relevant parameters of a novel glandless CSO sample under accelerated oxidative storage conditions. A common commercial r-CSO product was included for comparison/reference. The resulting data provided fundamental information on the storage stability of r-CSO. Furthermore, the patterns of change in those parameters over the storage period consistently indicated that c-CSO possessed higher oxidative stability than r-CSO. The presence of 50 times more tocopherols in c-CSO should be a contributing factor to the high degree of oxidation stability of c-CSO. Thus, in addition to nutrient values, c-CSO could be applied as an antioxidation additive to extend the shelf life of relevant food products (such as cottonseed or other plant-based butter products). This work also showed the potential of FTIR analysis as a non-invasive versatile technique for evaluating the oxidative degrees of CSO-containing products using a simple and fast method.

It should be noted that the accelerated oxidation storage technique was used to evaluate the oxidative stability of the novel c-CSO in comparison with r-CSO. This technique speeds up degradation processes; therefore, it is frequently used to assess the oxidative stability of food products [88]. However, the elevated temperature (i.e., 60 °C in this work) could lead to chemical mechanism changes and potential artifact formation [89]. Thus, some precautions would be necessary if the information is applied to normal and/or realistic storage conditions for CSO products.

## Figures and Tables

**Table 1:** Acid value (I_KOH_), peroxide value (PV) and p-anisidine value (pAV) of c-CSO and r-CSO samples impacted by accelerated storage time. Data are presented in the format of average ± SD (n = 3). Different letters in the same column indicate the values are significantly different at *p* ≤ 0.05.

	I_KOH_ (mg g^−1^)		PV (meq kg^−1^)		pAV	
Time (d)	c-CSO	r-CSO	c-CSO	r-CSO	c-CSO	r-CSO
0	2.89 ± 0.06 b	2.70 ± 0.01 c	59.31 ± 9.60 a	44.81 ± 8.84 a	0.12 ± 0.01 f	4.85 ± 0.80 b
5	3.17 ± 0.02 a	3.10 ± 0.07 b	45.09 ± 11.0 ab	51.75 ± 5.81 a	0.40 ± 0.005 e	4.57 ± 0.14 b
10	3.15 ± 0.24 a	3.31 ± 0.18 a	45.35 ± 8.45 ab	49.77 ± 10.3 a	0.59 ± 0.02 d	4.57 ± 0.05 b
20	3.21 ± 0.08 a	3.43 ± 0.07 a	61.56 ± 22.5 a	48.62 ± 2.70 a	0.73 ± 0.02 c	4.74 ± 0.09 b
30	3.14 ± 0.10 a	3.38 ± 0.03 a	49.92 ± 22.3 b	39.22 ± 20.1 a	1.08 ± 0.002 b	5.82 ± 0.06 a
45	3.21 ± 0.13 a	3.46 ± 0.06 a	22.94 ± 9.31 b	41.82 ± 8.54 a	1.62 ± 0.05 a	5.66 ± 0.03 a

**Table 2:** Absorptivity at 232 and 270 nm (E_232_ and E_270_) and ratio of absorptivity at 400 and 600 nm (E_4_/E_6_) of the c-CSO and r-CSO samples impacted by the accelerated storage time. Data are presented in the format of average ± SD (n = 3). Different letters in the same column indicate the values are significantly different at *p* ≤ 0.05.

	I_232_ (L g^−1^ cm^−1^) ^1^		I_270_ (L g^−1^ cm^−1^) ^2^		E_4_/E_6_ ^3^	
Time (d)	c-CSO	r-CSO	c-CSO	r-CSO	c-CSO	r-CSO
0	1.203 ± 0.077 b	3.413 ± 0.139 a	0.033 ± 0.002 d	0.168 ± 0.099 b	6.91 ± 0.77 c	9.06 ± 0.20 d
5	ND ^4^	ND	0.045 ± 0.004 d	0.181 ± 0.009 b	19.58 ± 1.00 b	10.65 ± 0.27 c
10	ND	ND	0.076 ± 0.002 c	0.206 ± 0.046 b	21.01 ± 2.63 b	11.69 ± 0.68 b
20	1.795 ± 0.246 a	3.303 ± 0.147 a	0.112 ± 0.016 b	0.249 ± 0.028 ab	23.01 ± 3.62 ab	10.91 ± 0.49 c
30	ND	ND	0.155 ± 0.009 a	0.301 ± 0.029 a	25.52 ± 1.00 a	13.57 ± 0.20 a
45	1.881 ± 0.307 a	3.451 ± 0.267 a	0.161 ± 0.009 a	0.338 ± 0.046 a	25.38 ± 1.31 a	13.94 ± 0.48 a

^1^ Measured with 1/700–1/5000 sample dilution. ^2^ Measured with 1/25–1/500 sample dilution. ^3^ Measured with undiluted samples. ^4^ Not determined.

## Data Availability

The original contributions presented in this study are included in the article. Further inquiries can be directed to the corresponding author.
